# Post-Anæsthetic Broncho-Pneumonia

**Published:** 1912-02-17

**Authors:** 


### POST-ANÆSTHETIC BRONCHO-PNEUMONIA.

### I.—Its Causation and Prevention.

Surgeons and anæsthetists are unfortunately familiar enough with the pulmonary complications which sometimes follow the administration of a general anæsthetic. Whether the severest complications are directly due to the anæsthetic, and the anæsthetic only, is still a matter of dispute, but it cannot reasonably be doubted that in the majority of cases certain pulmonary complications following a narcosis are rightly to be ascribed to the influence of the anæsthetic itself.

### Some Theories Advanced.

Various theories are advanced to explain this fact. It is held that during general anæsthesia the supply of oxygen to the lungs is interfered with, and the amount of blood traversing the lungs in a fully oxygenated condition is less than normal; consequently the nutrition of the delicate lung tissue is interfered with, its vitality is lowered, and the pneumonia bacillus, which is omnipresent almost, has a chance of settling and inducing the disease in the part less well nourished than normally. Another theory supposes that the anæsthetic itself directly lowers the temperature of the epithelium of the bronchioles and alveoli; that such cooling, again, lowers the vitality of this delicate tissue, and in this manner predisposes to the disease.

A further theory, and one more likely to be correct, holds that the lowering of the tissue vitality is dependent upon nervous influences for which the anæsthetic is responsible. Recent researches have tended to show that anæsthesia is caused by the temporary destruction of part of the protoplasm of ganglion cells—the so-called dissociation theory—and that this breaking down (or lipolytic action, since it is essentially the fatty element of the cell that is affected) sets free a certain amount of toxin which directly acts upon the tissue cells, decreasing their vitality and, in the case of the most delicate cells such as those which constitute the alveolar lung epithelium, destroying some of them under certain conditions.

### The Dissociation Suggestion.

It is impossible to discuss these theories at length, but it may be stated that the blood of an anæsthetised, or recently anæsthetised, person gives certain reactions analogous to the Wassermann reaction in syphilis, which strongly support the dissociation suggestion. The older theory that post-anæsthetic broncho-pneumonia is due solely to the exacerbation of an already existing capillary bronchitis is not tenable, although there is no doubt that the existence of bronchitis in a patient to be anæsthetised with a general anæsthetic renders the risk of subsequent broncho-pneumonia a probability, whereas in other cases where no previous bronchial condition exists it is merely a possibility.

Statistics compiled from many sources tend to show that pulmonary complications after a general anæsthetic are much more common than is generally supposed. A recent authority puts them as high as 80 per cent.; another investigator found that out of 300 cases no fewer than 260 showed pulmonary signs some time after the anæsthetic had been given. Every anæsthetist who has taken the trouble carefully to examine his cases systematically must have been struck with the large percentage in which râles, rhonchi, and crepitations were audible some time after the operation. It is true in the majority of cases these signs never grow into symptoms. They do not cause the patient any inconvenience, and they are only discovered on methodical routine examination. When they progress so that the temperature and the pulse-rate rise, and other symptoms make their appearance, these complications become of clinical importance, for the patient then suffers from broncho-pneumonia.

### The Pneumococcus.

Post-anæsthetic broncho-pneumonia is caused by the pneumococcus, and differs from the other forms of the disease only in degree. It is more common in old and enfeebled patients, in those who are bronchitic and emphysematous, and in those who are alcoholics; in the last class it is particularly severe. It is most common, undoubtedly, after abdominal operations, especially in those in which deep inspiration on the part of the patient is interfered with by tight abdominal bandaging, as in cases of ventral herniæ, and of operations on the liver or stomach. Usually it commences some hours after the patient awakes from the anæsthetic, as in bronchitis, with cough and frequent expectoration of white frothy phlegm. On examination râles and rhonchi are heard over the affected lung, and usually it is possible, even in the early stages, to find one or two spots where the expiration note is prolonged and where signs of consolidation are apparent. In mild cases only one or two such spots may be found; in severe cases the area of consolidation is much larger and the signs are much more obvious.

The condition, by reason of its manifest importance, has occasioned much discussion, and even now there is no consensus regarding its causation; while the views with regard to its prevention differ widely.

### Anæsthetist's Standpoint.

From the anæsthetist's point of view one or two facts stand out clearly. One is that the anæsthetic cannot always be blamed for the disease—in other words, that the title "post-anæsthetic broncho-pneumonia" is not altogether just. The surgeon's want of common-sense in needlessly injuring tissues and in unduly prolonging the operation is often more responsible for the onset of these pulmonary symptoms than the alleged want of care on the part of the anæsthetist. Another fact is that ether seems to be more commonly the cause of such symptoms, in cases where the only reasonably assignable cause is the anæsthetic, than chloroform. Some anæsthetists maintain that there is no great difference between these two anæsthetics so far as pulmonary after-effects are concerned. In Germany, where the ethyl chloroform is exclusively used, the percentage of cases of pulmonary complications after chloroform narcosis is not less than in this country, where both the ethylic and the cheaper methyl chloroform are employed.

Pneumonia has followed the administration of every variety of general anæsthetic and almost every known method.

### The Literature of the Subject.

When we review the literature we find that none of the prophylactic measures advised can be absolutely relied upon. The best and most reasonable is to ensure that the patient gets a sufficiency of oxygen *plus* carbonic acid—in fact, as near a mixture to ordinary atmospheric air as possible—and that this mixture, with the anæsthetic vapour, is given at an equable temperature. It is true that ordinarily a patient breathing a cold mixture of vapour and air warms this by the passage of the mixture across the surface of the nasal epithelium, which considerably raises the temperature of the air inspired.

The use of open methods for ether and chloroform administration has certainly tended to lower the percentage of pulmonary after-effects; the use of apparatus which ensures a minimum percentage of anæsthetic vapours being administered has further lowered the percentage. It is also claimed that the use of certain adjuvants, chief among which are morphine-scopolamine and omnopon, has also tended to decrease the number of cases of post-anæsthetic broncho-pneumonia.

### Scopolamine.

In the case of scopolamine experience has not shown this claim to be warranted; indeed some anæsthetists believe, with justification, that the employment of this adjuvant, by making the respiration shallower, directly predisposes to pulmonary after-effects. The opium derivatives are also said to do so, but the accusation is founded on less evidence in their case; the certainty is that the employment of these adjuvants enables the anæsthetist to use a relatively small dose of anæsthetic.

### A German Precaution.

Recently German authorities have advised, as a prophylactic precaution, the injection of certain drugs, such as quinine and salipin. It is claimed that by the routine use of these drugs as preliminaries the investigators have anæsthetised several hundred patients in succession without a single case of pulmonary complications "of any consequence" ensuing. French authorities do not find this precaution so useful, and it has not become popular.

A point of some importance is the free ventilation of the operating-theatre itself.

It is an undoubted fact that cases of broncho-pneumonia following operation are more frequent in institutions where the theatre is ventilated on the plenum system.

### Ventilation of the Theatre.

This has been pointed out in an interesting comparison between the results at the Hamburg Eppendorf Hospital, where the theatre is built out from the main wards and approached by a corridor which is open to the air on both sides, and the results obtained at other institutions where the operating theatre is an interior room with artificial ventilation. At Eppendorf the percentage of serious cases of broncho-pneumonia have sunk from 23 to 8.

By the intravenous injection of ether for anæsthetic purposes—a method much used on the Continent since 1910—it is claimed that all post-anæsthetic pulmonary complications are avoided. There is sufficient evidence already to show that this is not absolutely true, since several cases of serious complications have been reported.

### The Intravenous Method.

If the dissociation theory is true, it is difficult to account for this alleged immunity. The method must, however, still be considered on its trial, and it is certainly not likely that it will replace the older methods of general anæsthesia.

For the present the only safeguard against pulmonary complications following general anæsthesia is to employ the usual precautions which every anæsthetist should take as a method of routine, no matter what the operation may be or what kind of anæsthetic he uses.

*(To be continued.)*

